# The 2024 Brain Tumor Segmentation Challenge Meningioma Radiotherapy (BraTS-MEN-RT) dataset

**DOI:** 10.1038/s41597-026-06649-x

**Published:** 2026-01-27

**Authors:** Dominic LaBella, Katherine Schumacher, Michael Mix, Kevin Leu, Shan McBurney-Lin, Pierre Nedelec, Javier Villanueva-Meyer, David R. Raleigh, Jonathan Shapey, Tom Vercauteren, Kazumi Chia, Marina Ivory, Theodore Barfoot, Omar Al-Salihi, Justin Leu, Lia M. Halasz, Yury Velichko, Chunhao Wang, John P. Kirkpatrick, Scott R. Floyd, Zachary J. Reitman, Trey C. Mullikin, Eugene J. Vaios, Ulas Bagci, Sean Sachdev, Jona A. Hattangadi-Gluth, Tyler M. Seibert, Nikdokht Farid, Connor Puett, Matthew W. Pease, Kevin Shiue, Syed M. Anwar, Shahriar Faghani, Peter Taylor, Pranav Warman, Jake Albrecht, András Jakab, Mana Moassefi, Verena Chung, Rong Chai, Alejandro Aristizabal, Alexandros Karargyris, Hasan Kassem, Sarthak Pati, Micah Sheller, Nazanin Maleki, Rachit Saluja, Florian Kofler, Christopher G. Schwarz, Philipp Lohmann, Phillipp Vollmuth, Louis Gagnon, Maruf Adewole, Li Hongwei B, Anahita Fathi Kazerooni, Nourel H. Tahon, Udunna Anazodo, Ahmed W. Moawad, Bjoern Menze, Marius G. Linguraru, Mariam Aboian, Benedikt Wiestler, Ujjwal Baid, Gian-Marco Conte, Andreas M. Rauschecker, Ayman Nada, Aly H. Abayazeed, Raymond Huang, Maria Correia de Verdier, Jeffrey D. Rudie, Spyridon Bakas, Evan Calabrese

**Affiliations:** 1 Department of Radiation Oncology, Duke University Medical Center, Durham, NC USA; 2 Department of Radiation Oncology, SUNY Upstate Medical University, Syracuse, NY USA; 3 Center for Intelligent Imaging (ci2), Department of Radiology and Biomedical Imaging, University of California San Francisco (UCSF), San Francisco, CA USA; 4 Departments of Radiation Oncology, Neurological Surgery, and Pathology, University of California San Francisco (UCSF), San Francisco, CA USA; 5 Department of Neurosurgery, King’s College Hospital, London, UK; 6 School of Biomedical Engineering & Imaging Sciences, King’s College London, London, UK; 7 Guy’s and St Thomas’ NHS Foundation Trust, London, UK; 8 Department of Radiation Oncology, University of Washington/Fred Hutchinson Cancer Center, Seattle, WA USA; 9 Department of Radiology, Northwestern University, Evanston, IL USA; 10 Department of Radiation Oncology, Northwestern University, Evanston, IL USA; 11 Department of Radiation Medicine and Applied Sciences, University of California San Diego, La Jolla, CA USA; 12 Department of Radiology, University of California San Diego, La Jolla, CA USA; 13 Department of Bioengineering, University of California San Diego, La Jolla, CA USA; 14 Department of Neurological Surgery, Indiana University School of Medicine, Indianapolis, IN USA; 15 Department of Radiation Oncology, Indiana University, Indianapolis, IN USA; 16 Children’s National Hospital, Washington, DC USA; 17 George Washington University, Washington, DC USA; 18 Department of Radiology, Mayo Clinic, Rochester, MN USA; 19 Duke University Medical Center, School of Medicine, Durham, NC USA; 20 Sage Bionetworks, Seattle, USA; 21 University of Zürich, Zürich, Switzerland; 22 Artificial Intelligence Lab, Department of Radiology, Mayo Clinic, Rochester, MN USA; 23 MLCommons, Beaverton, OR USA; 24 Factored AI, Palo Alto, CA USA; 25 Center For Federated Learning in Medicine, Indiana University, Indianapolis, IN USA; 26 Division of Computational Pathology, Department of Pathology and Laboratory Medicine, Indiana University School of Medicine, Indianapolis, IN USA; 27 Medical Working Group, MLCommons, San Francisco, CA USA; 28 Intel Corporation, Santa Clara, CA USA; 29 Yale University, New Haven, CT USA; 30 Cornell University, Ithaca, NY USA; 31 Helmholtz AI, Helmholtz Munich, Oberschleißheim, Germany; 32 Department of Informatics, Technical University Munich, Munich, Germany; 33 TranslaTUM - Central Institute for Translational Cancer Research, Technical University of Munich, Munich, Germany; 34 Department of Diagnostic and Interventional Neuroradiology, School of Medicine, Klinikum rechts der Isar, Technical University of Munich, Munich, Germany; 35 Institute of Neuroscience and Medicine (INM-4), Research Center Juelich, Juelich, Germany; 36 Department of Nuclear Medicine, University Hospital RWTH Aachen, Aachen, Germany; 37 Department of Medical Image Computing, German Cancer Research Center (DKFZ), Heidelberg, Germany; 38 Department of Neuroradiology, University Hospital Bonn, Bonn, Germany; 39 Department of Radiology and Nuclear Medicine, Université Laval, Québec, Québec, Canada; 40 Medical Artificial Intelligence (MAI) Lab, Crestview Radiology, Lagos, Nigeria; 41 Athinoula A Martinos Center for Biomedical Imaging, Massachusetts General Hospital, Boston, MA USA; 42 Department of Neuroradiology, Technical University of Munich, Munich, Germany; 43 University of Zurich, Zurich, Switzerland; 44 Children’s Hospital of Philadelphia, University of Pennsylvania, Philadelphia, PA USA; 45 Center for AI and Data Science for Integrated Diagnostics (AI2D) and Center for Biomedical Image Computing and Analytics (CBICA), University of Pennsylvania, Philadelphia, PA USA; 46 University of Missouri, Columbia, MO USA; 47 Montreal Neurological Institute (MNI), McGill University, Montreal, QC Canada; 48 Mercy Catholic Medical Center, Darby, PA USA; 49 Biomedical Image Analysis and Machine Learning, Department of Quantitative Biomedicine, University of Zurich, Zurich, Switzerland; 50 Emory University, Atlanta, GA USA; 51 Neosoma Inc. Stanford Medicine, Stanford, CA USA; 52 Department of Radiology, Brigham and Women’s Hospital, Boston, MA USA; 53 Department of Surgical Sciences, Section of Neuroradiology, Uppsala University, Uppsala, Sweden; 54 Department of Radiology, University of California San Diego, San Diego, CA USA; 55 Department of Radiology and Imaging Sciences, Indiana University School of Medicine, Indianapolis, IN USA; 56 Department of Biostatistics and Health Data Science, Indiana University School of Medicine, Indianapolis, IN USA; 57 Duke University Medical Center, Department of Radiology, Durham, NC USA

**Keywords:** Translational research, Brain imaging, Scientific data

## Abstract

Meningiomas are the most common primary intracranial tumors, frequently requiring radiotherapy as a part of management. Effective radiotherapy planning for meningiomas necessitates accurate and consistent segmentation of target volumes on MRI, a process that is complex, labor-intensive, and dependent on expert expertise. The 2024 Brain Tumor Segmentation Challenge Meningioma Radiotherapy (BraTS-MEN-RT) Dataset addresses this problem by providing the largest multi-institutional collection of systematically annotated radiotherapy planning MRIs for meningiomas. Publicly accessible, this dataset comprises 570 radiotherapy planning 3D T1-weighted post-contrast MRIs at native resolutions, with 500 cases featuring expert-annotated gross tumor volumes (GTV). Annotations follow standardized radiotherapy planning protocols and include both intact and postoperative meningioma cases, ensuring wide clinical relevance. Contributions from seven diverse medical centers across the United States and the United Kingdom enhance the dataset’s generalizability. The dataset aims to accelerate the development of automated segmentation methods for radiotherapy planning, improving workflow efficiency, reducing interobserver variability, and ultimately enhancing patient outcomes.

## Background & Summary

Meningiomas are the most common intracranial tumors, encompassing approximately 37% of all primary central nervous system (CNS) neoplasms and up to 55% of non-malignant CNS tumors^1–3^. While the majority of these tumors (commonly WHO grade 1) are benign, higher-grade meningiomas (WHO grades 2 and 3) carry a significantly higher risk of recurrence and morbidity^4,5^. Management strategies for symptomatic meningioma generally involve maximal surgical resection when anatomically feasible, often followed by radiotherapy (RT) for higher-grade or incompletely resected disease^6–8^.

Despite wide adoption of RT in both the intact (unresected) and postoperative contexts, the process of defining the radiation target volume—namely the gross tumor volume (GTV) and surrounding tissues at risk—remains labor-intensive and highly reliant on specialized expertise. Established guidelines, such as those from EORTC 22042–026042 and RTOG 0539, detail the delineation of meningioma tumor beds on contrast-enhanced T1-weighted magnetic resonance tomography (MRI) for primary or adjuvant therapy^9–11^. However, segmenting meningiomas in real-world RT workflows is challenging: postoperative cavities may exhibit complex morphologies, postoperative changes, and artifact from adjacent hardware. In addition, some patients receive stereotactic radiosurgery (SRS) with dedicated headframes that can result in additional image artifacts^12,13^.

Although deep learning-based segmentation methods have seen considerable success for preoperative brain tumor imaging, relatively few solutions have targeted the unique requirements of RT planning^14–19^. Prior efforts—such as earlier Brain Tumor Segmentation (BraTS) challenges—commonly emphasize skull-stripped, isotropically resampled images to a standard anatomical space, along with multiple MRI sequences^19,20^. While valuable for diagnostic and predictive modeling purposes, these pre-processing steps can limit the direct clinical utility for RT, where native spatial resolution and inclusion of extracranial anatomy (e.g., stereotactic localizer fiducials, fixation devices) are essential^21–24^.

Herein, we introduce the 2024 Brain Tumor Segmentation Challenge Meningioma Radiotherapy (BraTS-MEN-RT) Dataset, a multi-institutional, expert-annotated resource encompassing 570 post-contrast T1-weighted (T1c) RT planning MRI scans for intact or postoperative meningiomas. Each study remains in its original resolution and orientation, with only defacing performed to preserve patient anonymity while retaining important extracranial structures^25–28^. A subset of 500 cases include corresponding GTV segmentations encompassing all meningioma lesions or at-risk resection cavities, harmonized according to established meningioma RT protocols^9–11^. This dataset is designed to facilitate the creation of clinically informed models that can effectively navigate the challenges posed by variability in image acquisition, SRS-specific artifacts, and postoperative imaging complexity.

In the sections that follow, we detail the data collection and annotation pipelines, describe robust quality assurance measures, and summarize the results from the BraTS-MEN-RT Dataset. By making this dataset freely available, we aim to accelerate the integration of automated segmentation algorithms into actual radiotherapy workflows, thereby improving the speed, consistency, and objectivity of meningioma RT planning.

## Methods

### Study population

The BraTS-MEN-RT Dataset comprises 3D T1c brain MRI scans obtained for clinical RT planning of meningiomas, including both intact and postoperative cases. Seven academic medical centers across the United States and the United Kingdom contributed. Participating institutions included Duke University, University of California San Francisco, State University of New York Upstate Medical University, University of Washington, University of Missouri, King’s College London, and University of California San Diego. Each institution retrospectively identified meningioma patients who underwent any modality of RT including Cobalt-60, external beam radiation therapy with photons, SRS, or proton therapy. Case inclusion criteria, whether diagnosis was made based on pathological, clinical, or radiological evidence, as well as case collection methods (e.g., random or consecutive sampling), were determined independently by each site, often leveraging pre-existing curated datasets developed for other purposes. For patients that underwent multiple courses of RT, each of their respective radiotherapy planning 3D T1c brain MRI scans could be included as a separate BraTS-MEN-RT case.

All centers contributing publicly accessible data adhered to their respective institutional review board (IRB) protocols ensuring compliance with ethical standards for research involving human subjects (DUKE IRB approval number: Pro00110695; MISS IRB approval number: 2096253 MU; SUNY Upstate IRB approval number: 2183481-1; UCSD IRB approval number: 809620; UCSF IRB approval number: 18-24633; UW Human Subjects Division IRB approval number: STUDY00020442). A waiver for informed consent was provided by each institution’s respective IRB. To protect patient confidentiality, all metadata within Digital Imaging and Communications in Medicine (DICOM) files were de-identified by each participating institution and reviewed to ensure that all patient identifiers were removed or anonymized before data release.

### Imaging data

**Fig. 1:**
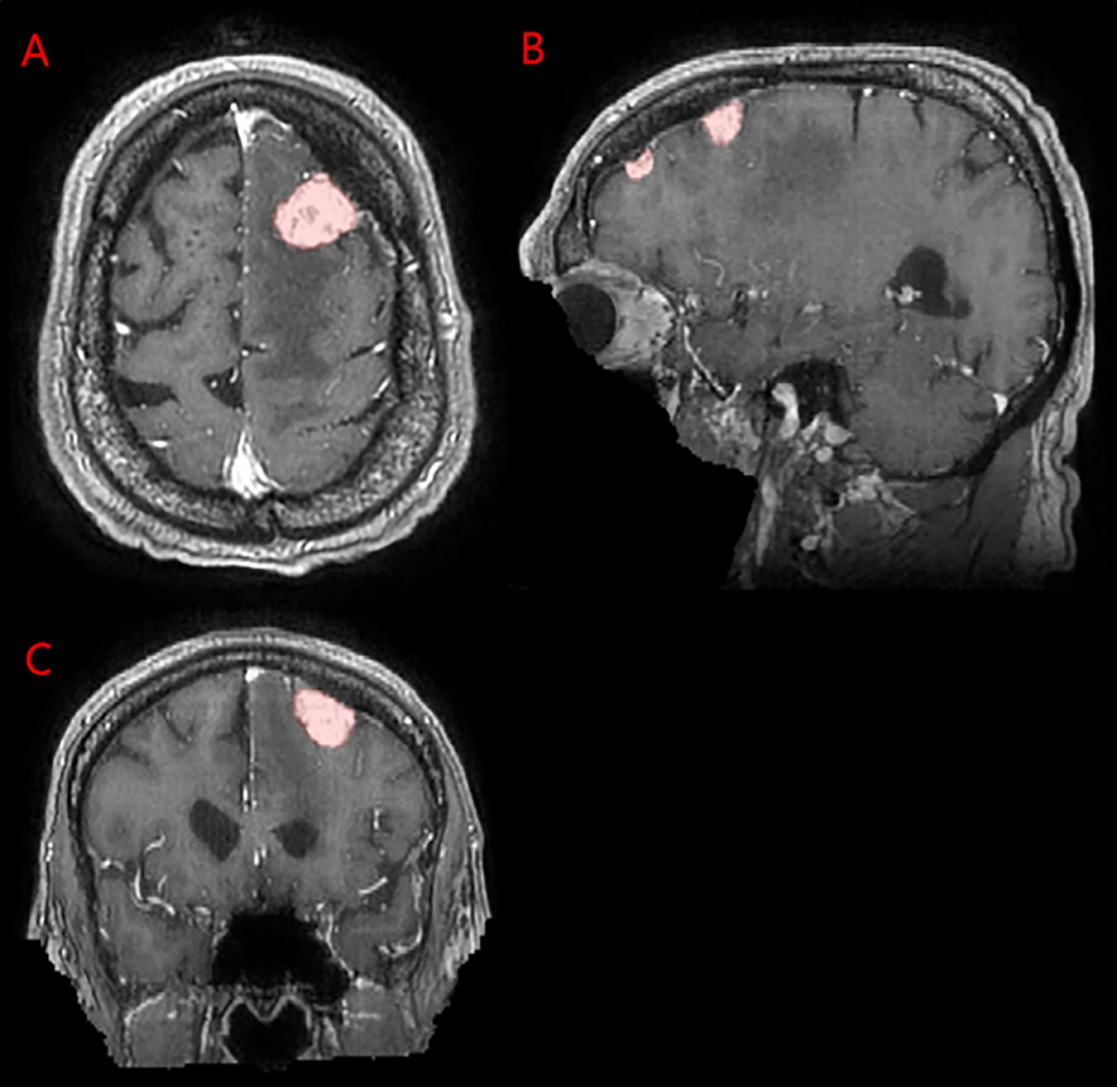
Panels A, B, and C depict two distinct intact meningiomas (red) on axial, sagittal, and coronal images respectively.

**Fig. 2:**
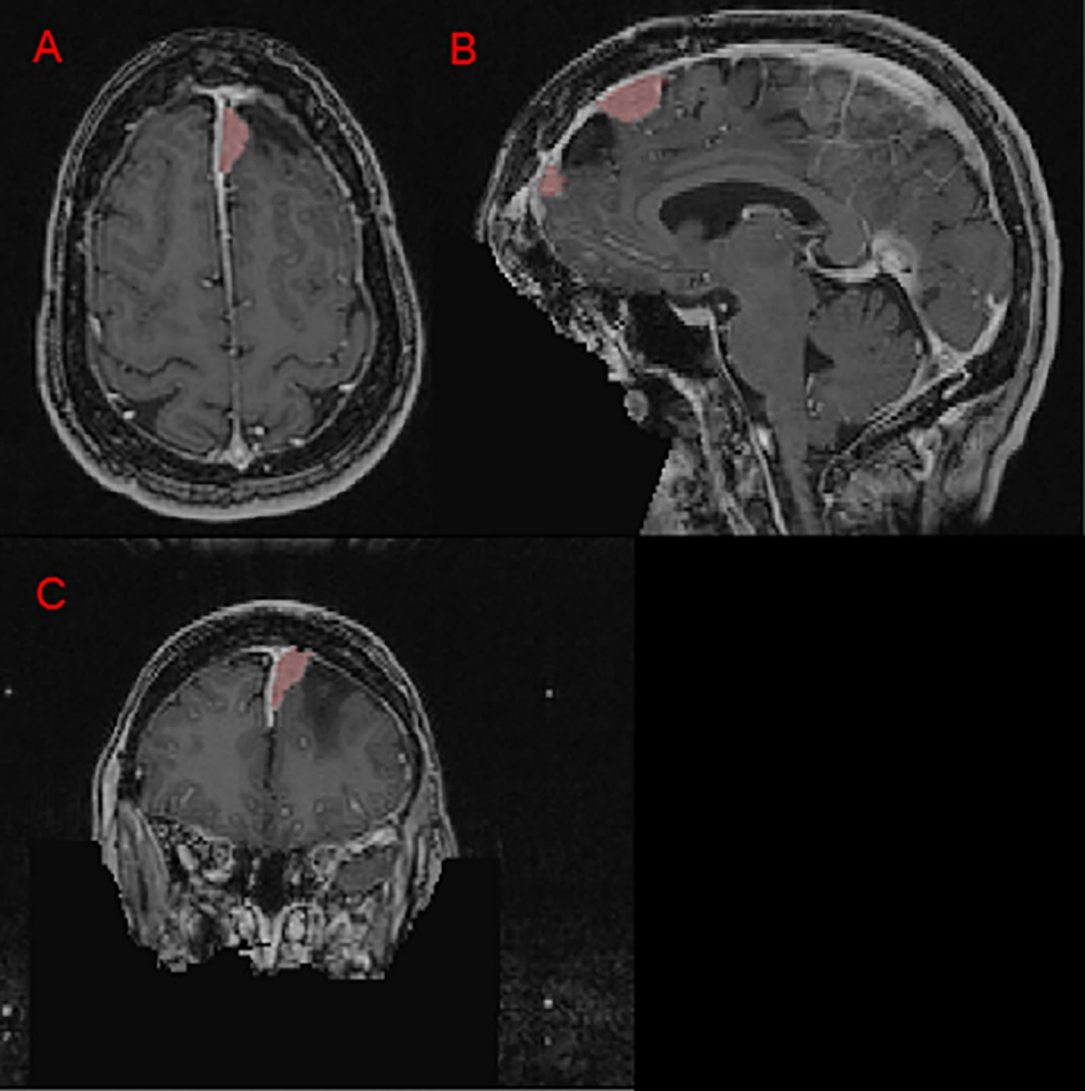
Panels A, B, and C depict a left anterior falcine meningioma (red) on axial, sagittal, and coronal images respectively. Panel B demonstrates an area of hypointense encephalomalacia between two separate anterior falcine meningioma components. Panels B and C highlight the defacing process, which removes pixels in and around the face to eliminate potentially identifying facial features.

**Fig. 3:**
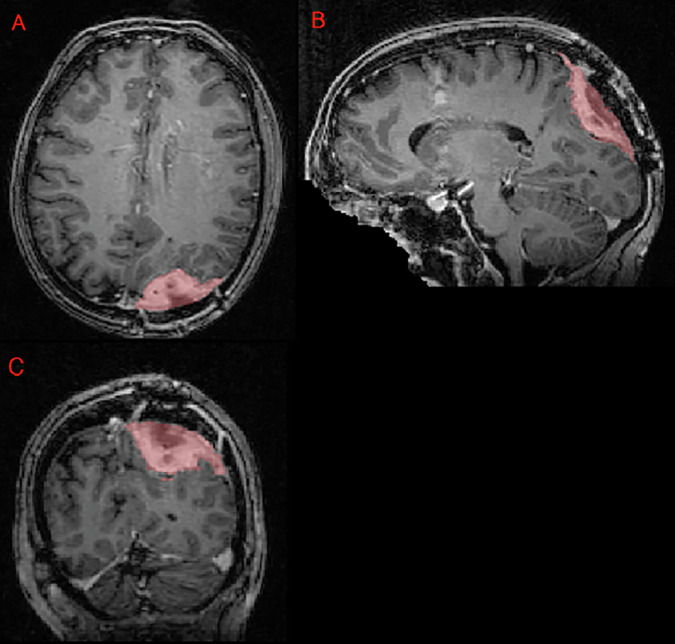
Panels A, B, and C showing a postoperative left parietal meningioma target volume (red) in the axial, sagittal, and coronal planes, respectively, as delineated by the treating institution.

The imaging dataset includes exclusively 3D T1c brain MRI scans in native acquisition resolution in either the intact (Fig. 1) or postoperative setting (Fig. 2, 3), which mimics the data available for most radiotherapy planning. While additional sequences such as T1-weighted, T2-weighted, T2-FLAIR, and computed tomography are sometimes used in clinical workflows, these were not consistently available nor required for RT planning at all centers and were therefore not included in the dataset.

Participating sites exported physician-delineated gross tumor volumes (GTVs) from their available DICOM-RT structure sets when present. Imaging parameters including vendor, scanner model, magnetic field strength, series description, sequence identifiers (sequence name, scanning sequence, sequence variant), repetition and echo times (TR, TE), inversion time (TI), flip angle, in-plane resolution (pixel spacing), slice thickness, acquisition and reconstruction matrices, pixel bandwidth, and coil name; varied substantially both within and across contributing institutions. Complete parameter metadata were available for a subset of cases, reflecting the diversity of acquisition protocols in clinical practice. To minimize barriers to data sharing and encourage participation, documentation of these imaging parameters was not mandated. This variability and partial availability of detailed imaging parameters underscore the dataset’s realism and enhance its value for developing and benchmarking automated segmentation models that must generalize across heterogeneous imaging conditions.

### Data splits

A total of 750 radiotherapy planning MRI exams from 747 unique patients were included in the BraTS-MEN-RT Dataset. These were divided into a training set (500/750, 66.7%), a validation set (70/750, 9.3%), and a private testing set (180/750, 24%). As feasible, the data splits were stratified by site to ensure balanced representation and consistency. In cases where there were multiple exams from a single patient, all such exams were included in only a single split. The University of Missouri data were included in the validation and testing set only, and the King’s College London data were included in the testing set only, due to the timing of data availability relative to the BraTS-MEN-RT Dataset release dates^29^. The 570 radiotherapy planning 3D T1c MRIs from the training and validation datasets, and expert-annotated GTV from the 500 training dataset cases are publicly available as part of this manuscript. The 180 cases from the testing dataset remain private to facilitate unbiased evaluation of new automated segmentation methods.

### Clinical data

**Fig. 4:**
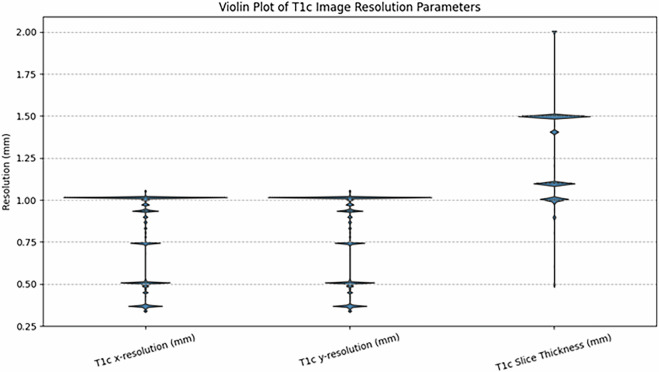
Violin plot depicting the distribution of T1c image resolution parameters (x-resolution, y-resolution, and slice thickness) from the BraTS-MEN-RT Dataset. Each violin shape represents the density distribution of the resolution measurements, with wider sections indicating higher frequency of measurements at specific resolution values.

Clinical-pathologic information, including patient age at the time of imaging, sex, and WHO tumor grade, was obtained from the respective electronic medical records at each contributing institution when possible. The age range was 11–90 years (including private testing set data) and 13–90 years in the publicly available data. The male to female ratio was 229:482 (including private testing set data) and 181:360 in the publicly available data. WHO grade was available in 342 of the 750 total cases (including private testing set data) and in 271 of the 570 publicly available cases. For training and validation cases, image x-resolutions and y-resolutions ranged from 0.338 to 1.055 mm, and slice thickness ranged from 0.488 to 2.000 mm, as shown in Fig. 4. Aggregate clinical and demographic data for the dataset are summarized in Table 1, including training, validation, and private testing data splits. Individual case-level data for the publicly available training and validation cases are publicly available on the Synapse data repository^29^.

**Table 1:** Basic clinical and demographic data for the BraTS-MEN-RT Dataset including the private test set data for cases with available patient demographic data.

Site	Total Cases	Training Set	Validation Set	Testing Set	Age (Median; Min-Max)	Male:Female	Grade 1	Grade 2	Grade 3
All Sites	750	500 (66.7%)	70 (9.3%)	180 (24%)	60 (11–90)	215:451	191	107	21
UCSF	225	180 (80%)	16 (7%)	29 (13%)	54 (11–88)	69:121	108	67	15
SUNY	189	152 (80%)	14 (7%)	23 (13%)	60 (16–90)	61:128	20	11	1
UW	128	101 (79%)	9 (7%)	18 (14%)	61.5 (25–83)	35:93	27	6	0
MISS	75	0 (0%)	25 (33%)	50 (67%)	63 (36–90)	21:54	7	6	1
DUKE	56	45 (80%)	4 (7%)	7 (13%)	61.5 (25–85)	21:35	26	12	2
KCL	49	0 (0%)	0 (0%)	49 (100%)	56 (31–79)	14:31	11	12	0
UCSD	28	22 (79%)	2 (7%)	4 (14%)	61 (20–87)	8:20	3	5	2

The data are categorized by the total cohort, as well as by contributing institution. Site abbreviations are as follows: DUKE (Duke University), SUNY (State University of New York), UW (University of Washington), MISS (University of Missouri), UCSF (University of California San Francisco), KCL (King’s College London), and UCSD (University of California San Diego).

### Image data pre-processing

**Fig. 5:**
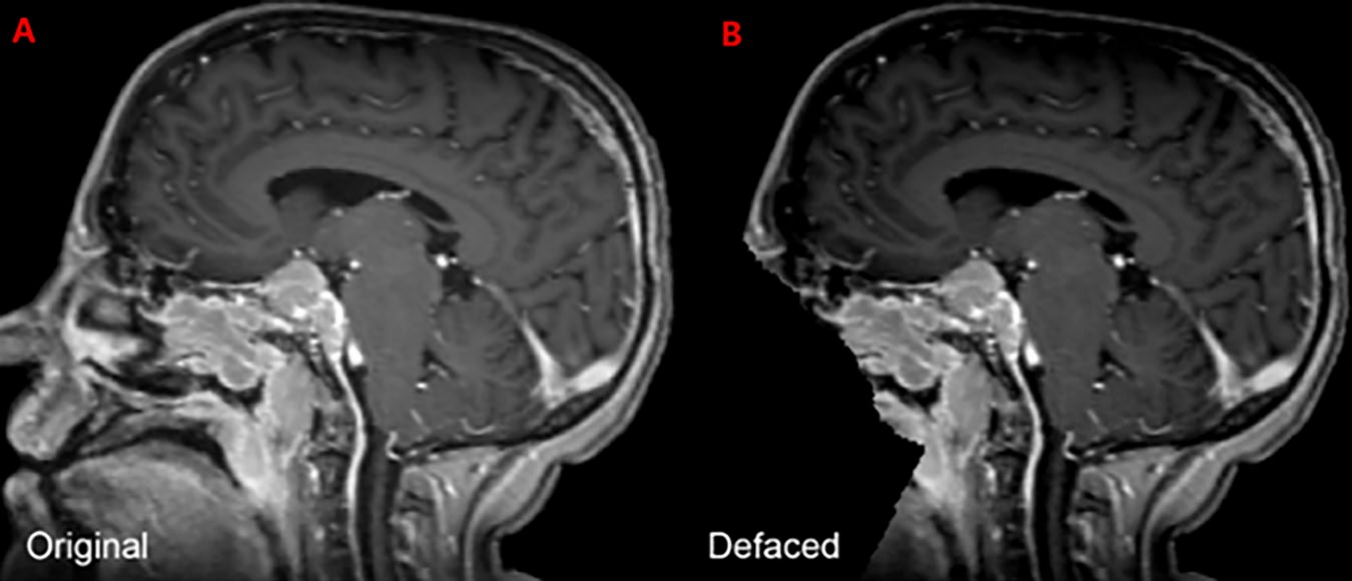
Example of a sagittal image of a brain MRI from the BraTS-MEN-RT training set, both before (**A**) and after (**B**) automated defacing.

Following anonymized DICOM-RT data collection from each respective institution, the raw DICOM-RT data were converted to Neuroimaging Informatics Technology Initiative (NIfTI) format using open-source software^30^. This process ensured that each MRI scan was accurately captured in its native spatial orientation and voxel resolution without any intensity resampling. To preserve extracranial anatomy and anatomical structures relevant to RT (e.g., stereotactic headframes, fixation devices), no skull-stripping was applied^22–24^. Instead, patient facial features were removed using the Analysis of Functional NeuroImages (AFNI) automated defacing algorithm with AFNI’s default parameters^26–28^. The AFNI defacing process eliminates patient-identifiable facial structures while retaining the rest of the cranial and extracranial volume as shown in Figs. 2C, 5.

**Fig. 6:**
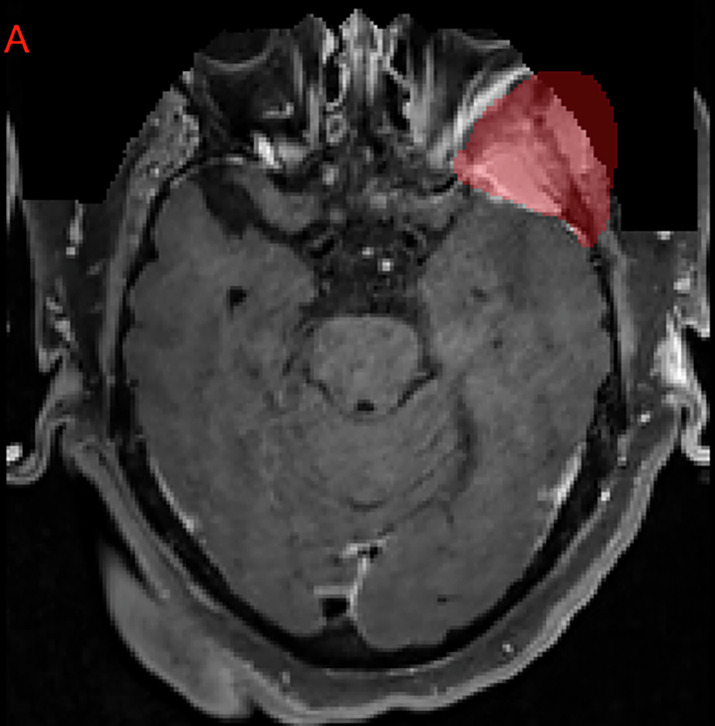
Example of an axial MRI image-label pair slice where the treating institution’s meningioma GTV extended outside of the defaced image. Note that this left sphenoid meningioma involves the skull base and extents extracranially into the left masticator space. This case was ultimately excluded from the challenge dataset due to the excessive volume within the anonymized region that would have needed to be reintroduced.

**Fig. 7:**
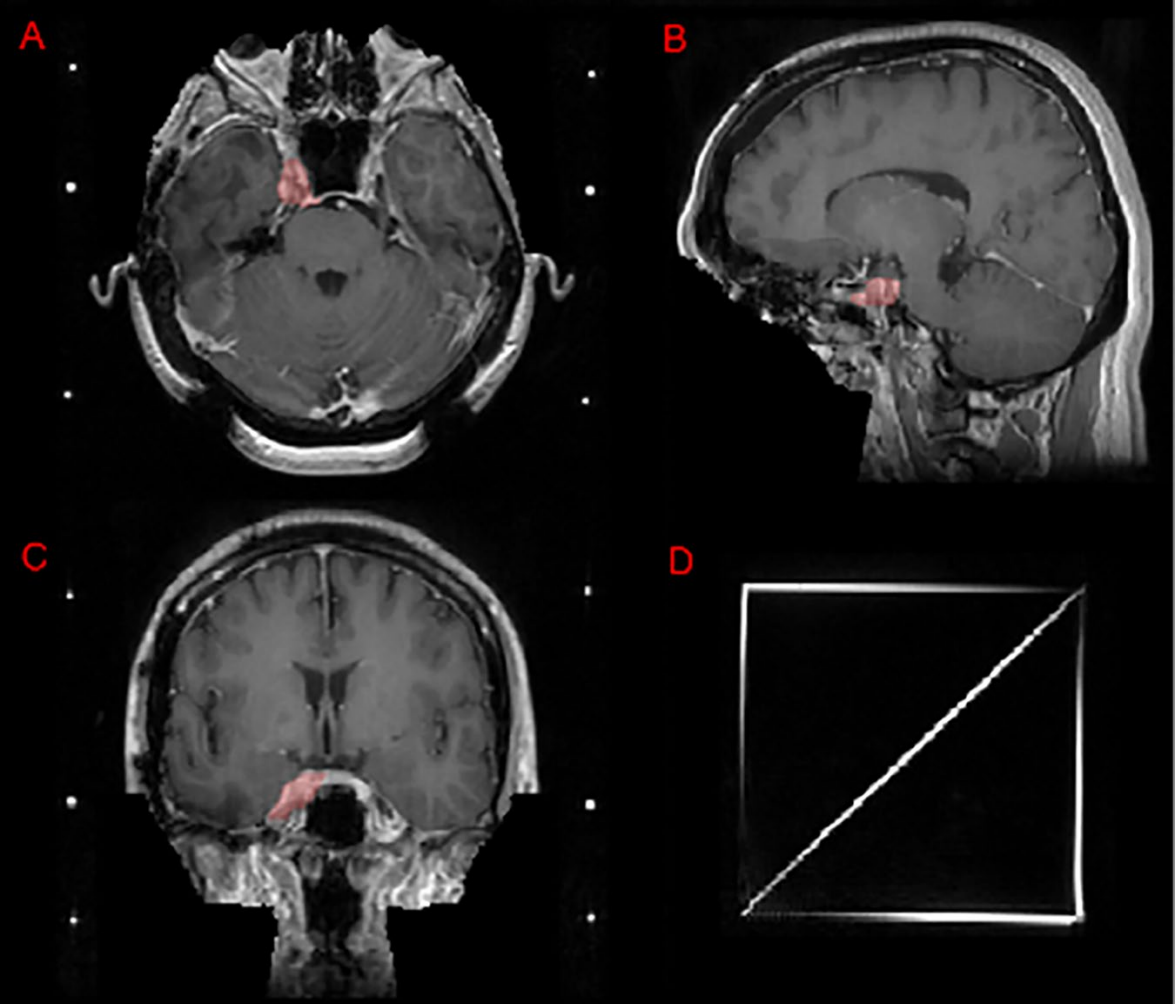
Image panels depicting a case that utilizes a SRS planning Gamma Knife headframe. Panels A, B, and C depict an intact meningioma (red) in the right Meckel’s cave on T1c axial, sagittal, and coronal images, respectively. Note that this challenge’s defacing technique preserves this meningioma as compared to a skull-stripping pre-processing technique which would have excluded at least part of this lesion. Panel D shows the SRS localizer box fiducials attached to a standard Gamma Knife headframe.

After defacing was performed, a robust quality-control procedure was employed, whereby each converted and defaced MRI volume underwent manual slice-by-slice review using ITK-SNAP to confirm the completeness of the intracranial volume and ascertain that no meningioma tissue was inadvertently removed^31^. Cases with meningiomas extending beyond the region of the face, such as anterior skull base tumors, were closely inspected to ensure no tumor signal was truncated. If partial tumor removal was detected, manual post-processing was performed to add back intensity to clinically appropriate brain MRI voxels corresponding to tumor (when partially removed) or exclude the case (if fully removed). Figure 6 demonstrates the partial inclusion of an institutional GTV on the defaced MRI, and Fig. 7 demonstrates a case of a patient that underwent SRS to an intact meningioma in Meckel’s cave that would have been at least partially excluded by a skull-stripping approach.

### Target volume definition

For each case, the binary segmentation consisted of a GTV segmentation corresponding to intact and/or postoperative meningioma. GTVs included areas of high-risk disease composed of any enhancing component of tumor on T1c MRI, including nodular dural tails when relevant, and the resection cavity for post-surgical cases^9–11^. Residual enhancing tissue in postoperative scans was considered part of the GTV if clinically determined to represent tumor.

In some patients with multiple intracranial meningiomas, the GTV segmentation included all visible meningiomas, regardless of whether every lesion had been clinically targeted in the respective case’s actual RT plan. This approach provides a comprehensive representation of meningioma burden and ensures that automated segmentation algorithms trained on these data are robust to multi-lesion scenarios.

These target volume definitions are clinically useful in radiotherapy planning and were agreed upon by a coalition of BraTS organizers consisting of board-certified radiation oncologists and board-certified fellowship trained neuroradiologists. The target volume segmentation definitions were agreed upon after review of the EORTC 22042-026042 and RTOG 0539 annotation protocols^9–11^.

### Annotation protocol

Each participating center had the option to provide their own clinical workflow GTV segmentations from the DICOM-RT structure sets. These institution-submitted segmentations served as an initial reference if they followed standard meningioma RT guidelines^9–11^. When institutional GTVs were either unavailable or did not conform to the challenge-defined annotation protocol, a deep convolutional neural network-based automated segmentation model was applied to generate a preliminary GTV mask. This model, built using the nnU-Net framework, was initially trained on the BraTS Preoperative Meningioma (BraTS-MEN) challenge data, which is the largest multi-institutional expert annotated multilabel preoperative meningioma multi-sequence MR image dataset to date^15,18,32–34^. Only T1c images and the tumor core segmentation (including enhancing and non-enhancing tumor) from the 1000 publicly available BraTS-MEN Dataset cases were utilized for nnU-Net training to most closely resemble the image and label data included in the BraTS-MEN-RT challenge. Training was performed using the default nnU-Net hyperparameters and data augmentation for a total of 1000 epochs^33^. Despite fundamental differences in processing (i.e., skull-stripping and atlas registration in the 2023 BraTS-MEN dataset), iterative retraining on accumulating BraTS-MEN-RT samples steadily improved the model’s ability to identify postoperative and residual/recurrent tumors. No formal quantitative analysis of nnU-Net training performance was conducted throughout the iterative re-training process, as the primary purpose was to accelerate data labeling.

**Fig. 8:**
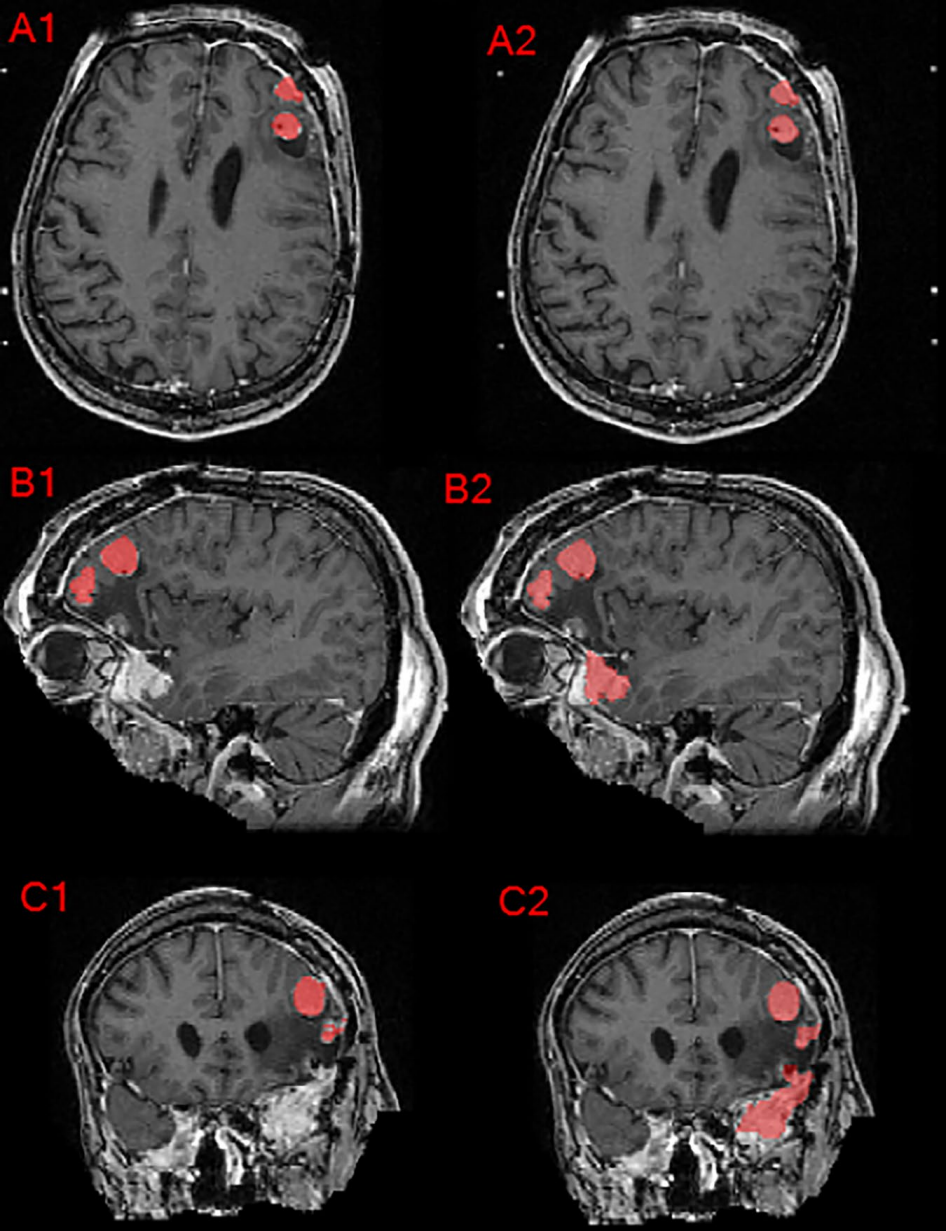
Axial, sagittal, and coronal brain MRI of a patient with multiple meningioma demonstrating the difference between the provided institution’s GTV as seen in panels A1, B1, and C1 compared to the manually revised target label as seen in panels A2, B2, and C2. Note that this case’s corrections involved inclusion of additional meningioma, correction of label edges, and inter-axial slice label smoothening.

After initial institution GTV collection or automated presegmentation of GTV, a senior radiation oncology resident (D.L.) conducted an initial review and potential revision through a slice-by-slice evaluation (ITK-SNAP) of each GTV and revised as needed to adhere to the BraTS-MEN-RT target volume definition as shown in Fig. 8^31^. During the initial revision phase of nnU-Net automated segmentation GTV, common edits included the following:

1. Adding or removing dural tail areas.
2. Modifying segmentation edges to conform accurately with enhancing tissue boundaries in postoperative meningioma.
3. Adding additional meningioma tumors outside of the clinical RT treatment volumes.

After initial revision, a board-certified attending neuroradiologist (E.C.) reviewed the corrected GTV for final approval.

This protocol, guided by clinical best practices in meningioma delineation, aimed to ensure segmentation consistency across institutions and tumor subtypes^7,9–11,35,36^.

## Data Records

The BraTS-MEN-RT Dataset training data (500/750, 66.7%), validation data (70/750, 9.33%), and clinical metadata are publicly available on Synapse at 10.7303/syn59059779^29^. The testing data (180/750, 24%) will be kept private for the foreseeable future to allow for unbiased evaluation of algorithms developed in future challenges including the MICCAI 2025 Lighthouse Challenge: “BraTS-Meningioma-RT: Meningioma Radiotherapy Segmentation”^30^. The “Meningioma radiotherapy supplementary clinical data and imaging parameters for training and validation sets.xlsx” file on the Synapse data repository describes the case level clinical patient data and the available image parameters for the training and validation cases^29^. The supplementary file “BraTS-MEN-RT Dataset Access Steps” provides step by step instructions on how to access the training image and segmentation data, validation image data, and the clinical patient data. All publicly available images, labels, and clinical metadata are of the CC-BY-NC license per Synapse policy.

## Technical Validation

### Patient clinical and demographic data

The clinical characteristics of individuals included in the BraTS-MEN-RT Dataset were sourced from medical records at each participating academic institution. Specific details regarding the methods used for data collection at each site were not disclosed, an approach intended to promote broader data contribution. The raw clinical data included in the BraTS-MEN-RT Dataset were not subjected to further independent validation.

### Image pre-processing and defacing

All images that underwent defacing using AFNI were manually reviewed by a fellowship-trained attending neuroradiologist (E.C.) and senior radiation oncology resident (D.L.) to ensure adequate defacing, presence of at least one intracranial meningioma or postoperative target, and absence of a non-meningioma intracranial tumor. Any pre-processing defacing errors were manually corrected before inclusion in the dataset. It is important to note that the AFNI defacing we used may have limitations. Its face coverage is similar to older programs like mri_deface and PyDeface, and studies have shown that replacing more of the face, and especially the eyebrow ridge, can potentially result in greater privacy protection albeit at the cost of greater patient anatomy elimination^21,22,37^. Future challenges should compare additional face anonymization methods, including algorithms that either replace facial features with synthetic faces or further expand the defacing mask while preserving tumor boundaries to reduce potential re-identification risk while preserving clinically relevant anatomy.

### Meningioma segmentations

All GTV segmentations were manually reviewed by a board-certified attending neuroradiologist “approver” (E.C.). In cases where the approver identified an inaccurate or incomplete segmentation, the case was further refined (E.C.) until satisfactory.

## Supplementary information

Supplementary Information BraTS-MEN-RT Data Resource Paper Data Access Steps

## Data Availability

The BraTS-MEN-RT Dataset image data, label data, and clinical metadata are publicly available on Synapse at 10.7303/syn59059779^29^.

## Code availability

In line with the scientific data principles of findability, accessibility, Interoperability, and reusability^38^, the tools used throughout the generation of these data are publicly available. Specifically, we used the dcmrtstruct2nii repository for conversion of DICOM-RT to NIfTI, which is publically available at (https://github.com/Sikerdebaard/dcmrtstruct2nii)^30^. We used the Analysis of Functional NeuroImages (AFNI) automated defacing algorithm for defacing, which is publically available at (https://github.com/afni/afni)^26,27^. The nnU-Net model as used for initial pre-automated segmentation and iterative re-pre-automated segmentation is publicly available at https://github.com/ecalabr/nnUNet_models^39^. Additional GTV segmentation modification code used for revision of some institution provided GTVs is publicly available as CC-BY 4.0 at https://github.com/dlabella29/BraTS-MEN-RT_Data_Resource^40^.
