# Supplementary material for: The 2024 Brain Tumor Segmentation Challenge Meningioma Radiotherapy (BraTS-MEN-RT) dataset
Source: Sci Data. 2026 Jan 27;13:306. doi: 10.1038/s41597-026-06649-x (PMC12948943; doi:10.1038/s41597-026-06649-x)
Supplement: Supplementary file 1 — Supplementary Information BraTS-MEN-RT Data Resource Paper Data Access Steps [file 41597_2026_6649_MOESM1_ESM.pdf]

This document outlines the steps needed to freely access the challenge restricted *BraTS Meningioma Radiotherapy Segmentation Dataset* (BraTS-MEN-RT)

Option 1:

1. Click on either of the following links, which will direct you to the Synapse hosting site.
    a. https://www.synapse.org/Synapse:syn59059779
    b. https://doi.org/10.7303/syn59059779

2. Click on the "key" symbol next to "Request Access" to login to Synapse.

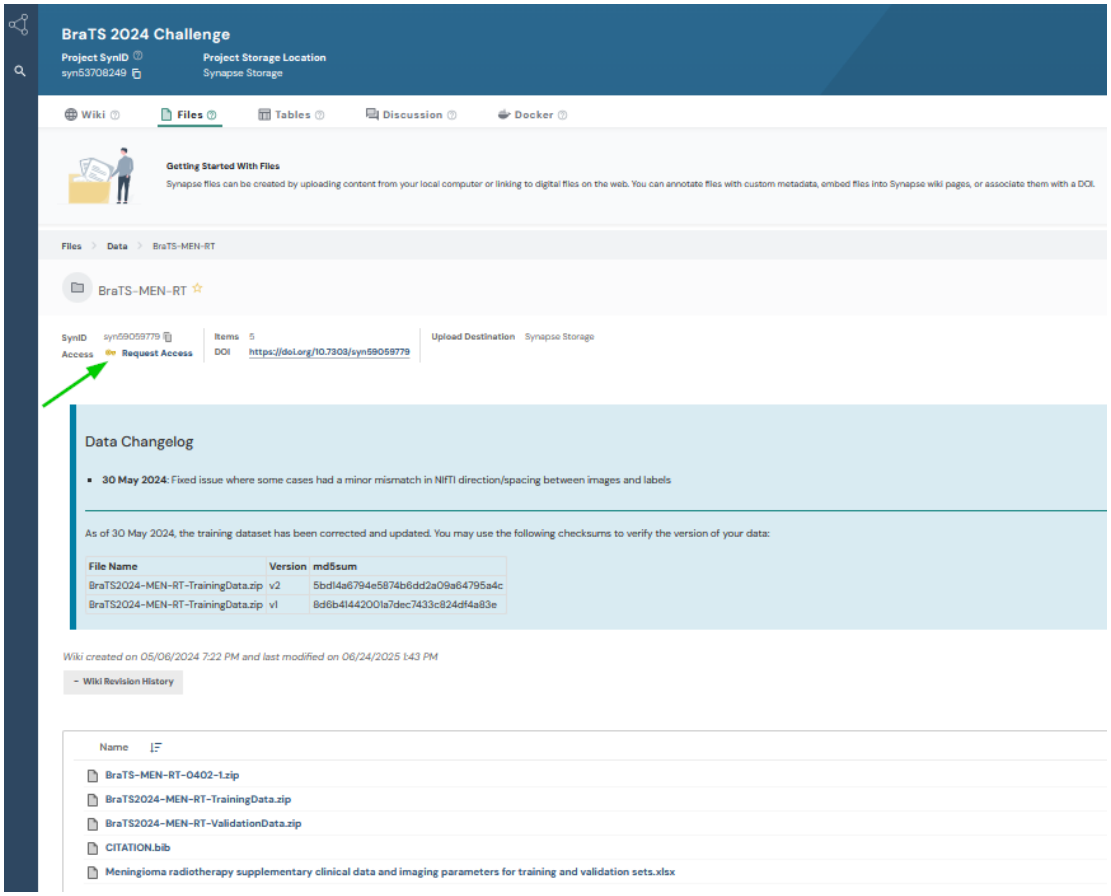

3. Either login to your previously created account or create an account.

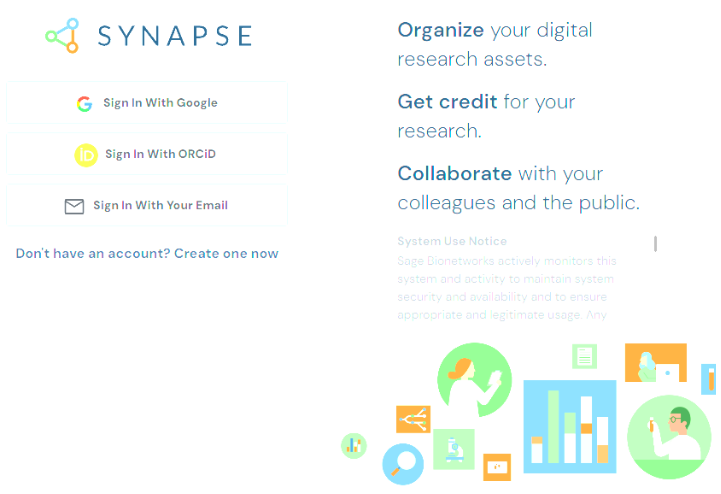

4. Accept the Terms of Use. This is required by the host site Synapse.

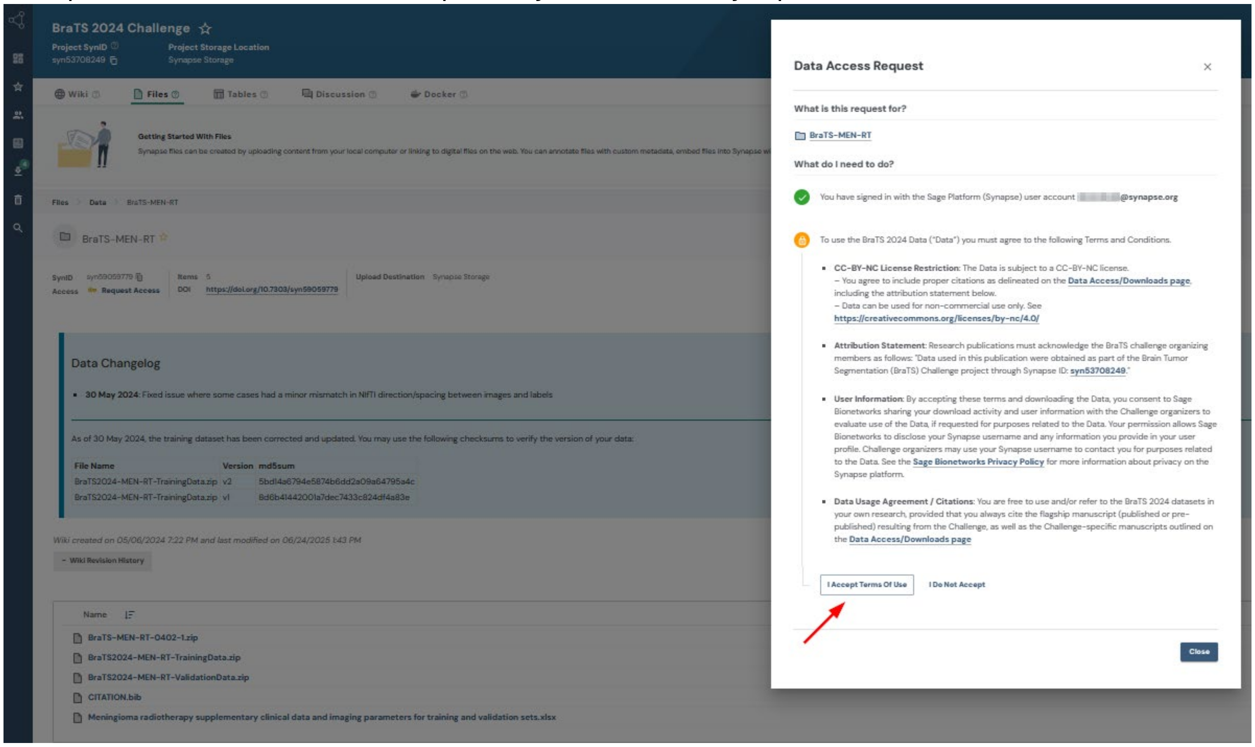

5. After logging in and accepting the Terms of Use, refresh the page. The “key” will change to a green “unlocked” symbol. Click on the download button for any of the respective files or folders.

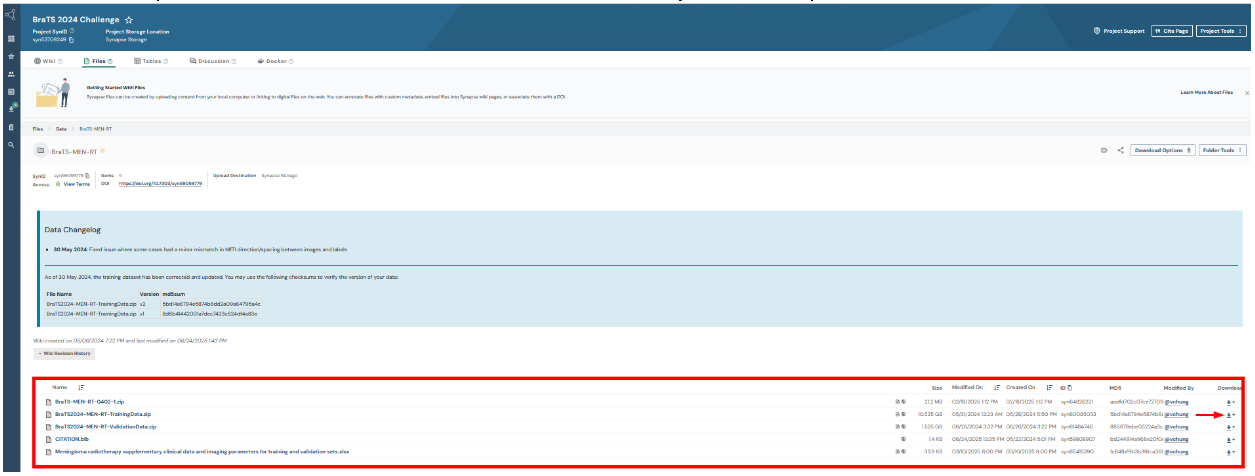

6. Click on the View Download List

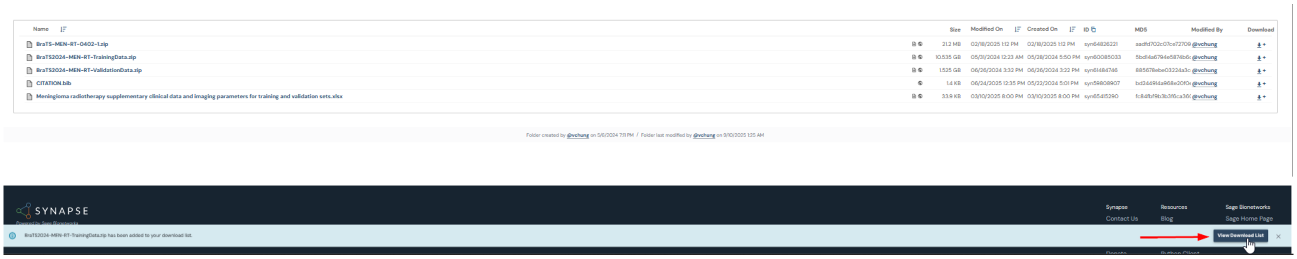

7. Accept the “Data-Specific Terms of Use”. This additional layer of restriction is required by Synapse for post-challenge access.

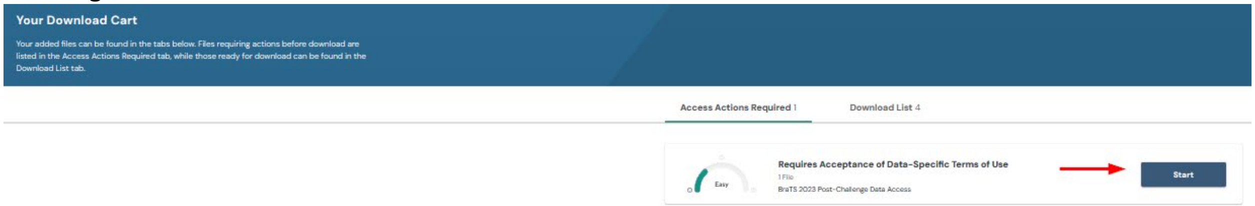

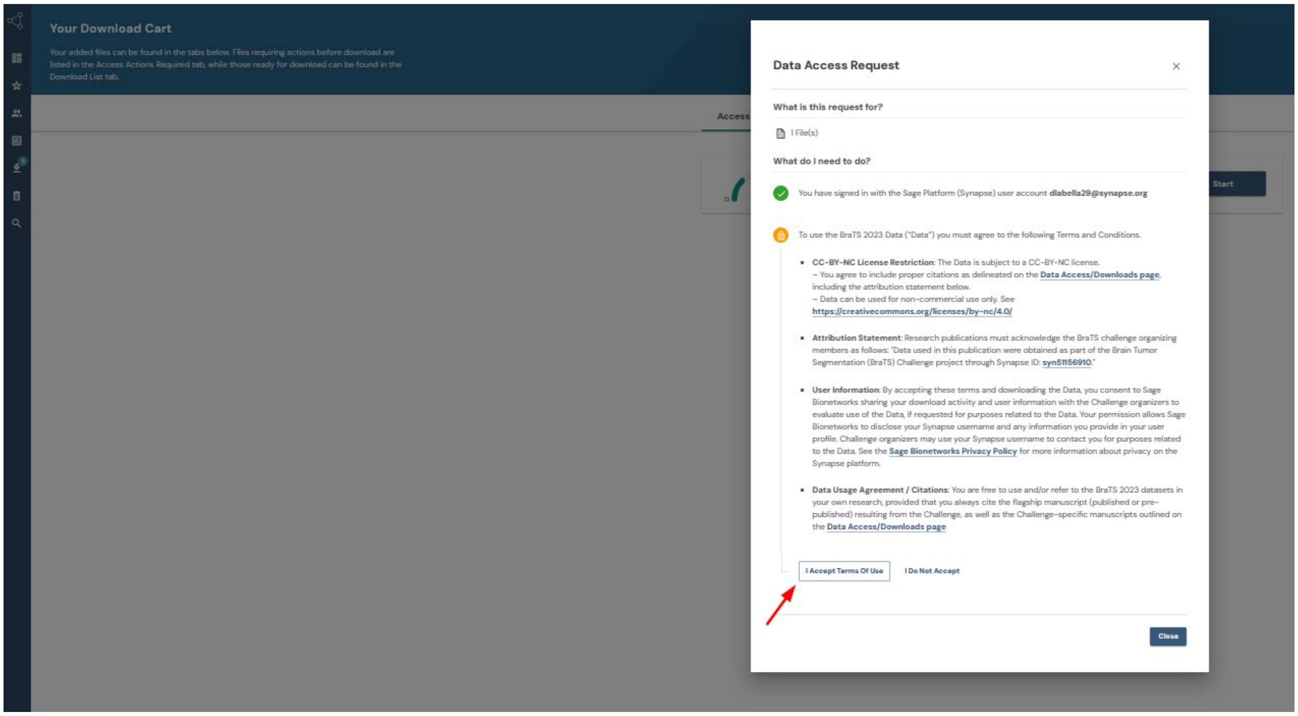

8. This will take you to the download cart. At which point you can click on the download button to download the data.

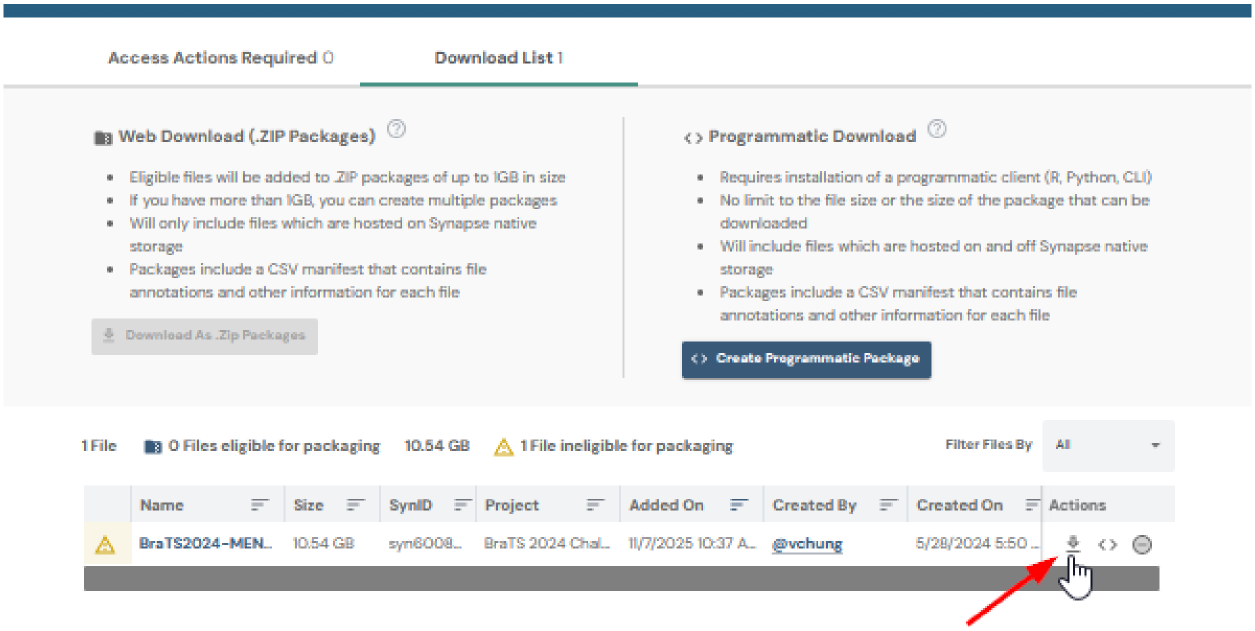

Option 2:

1. After navigating to the Synapse Repository page as seen in Option 1, click on the file of interest to get to its details page.

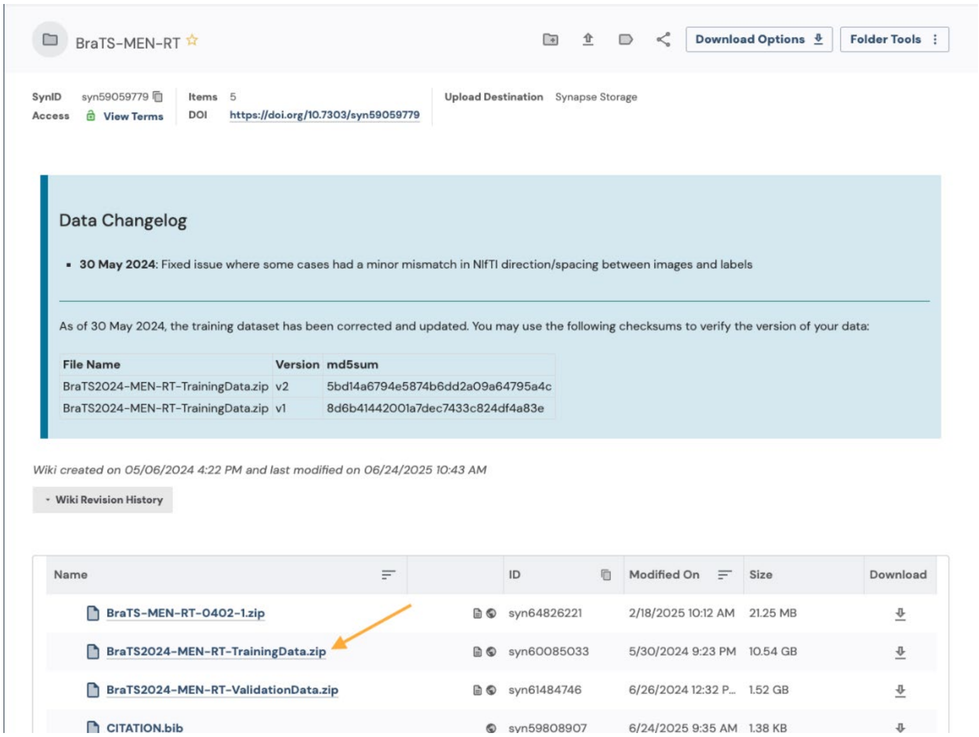

2. Click on **Download Options** button in the top-right corner.

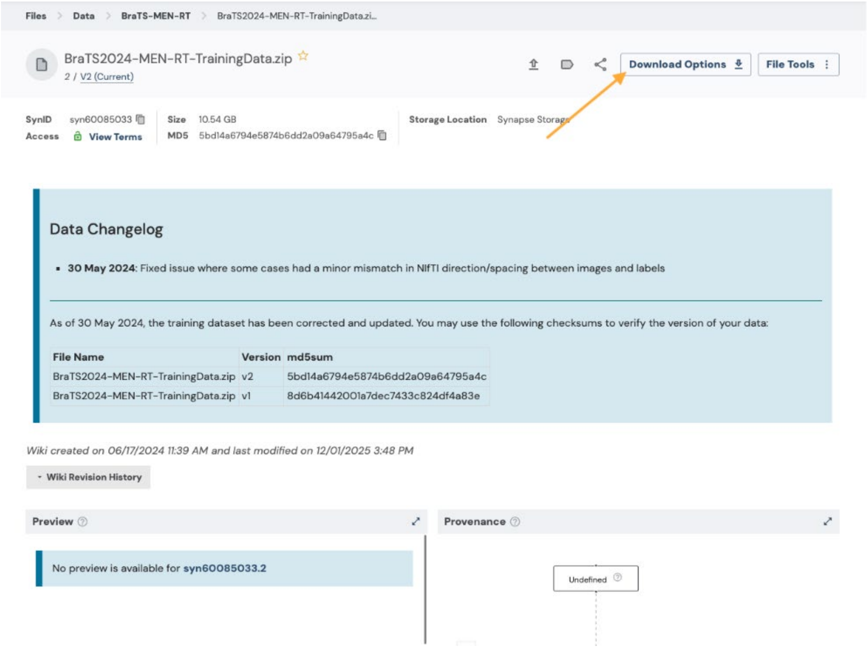

3. Click **Download File**.

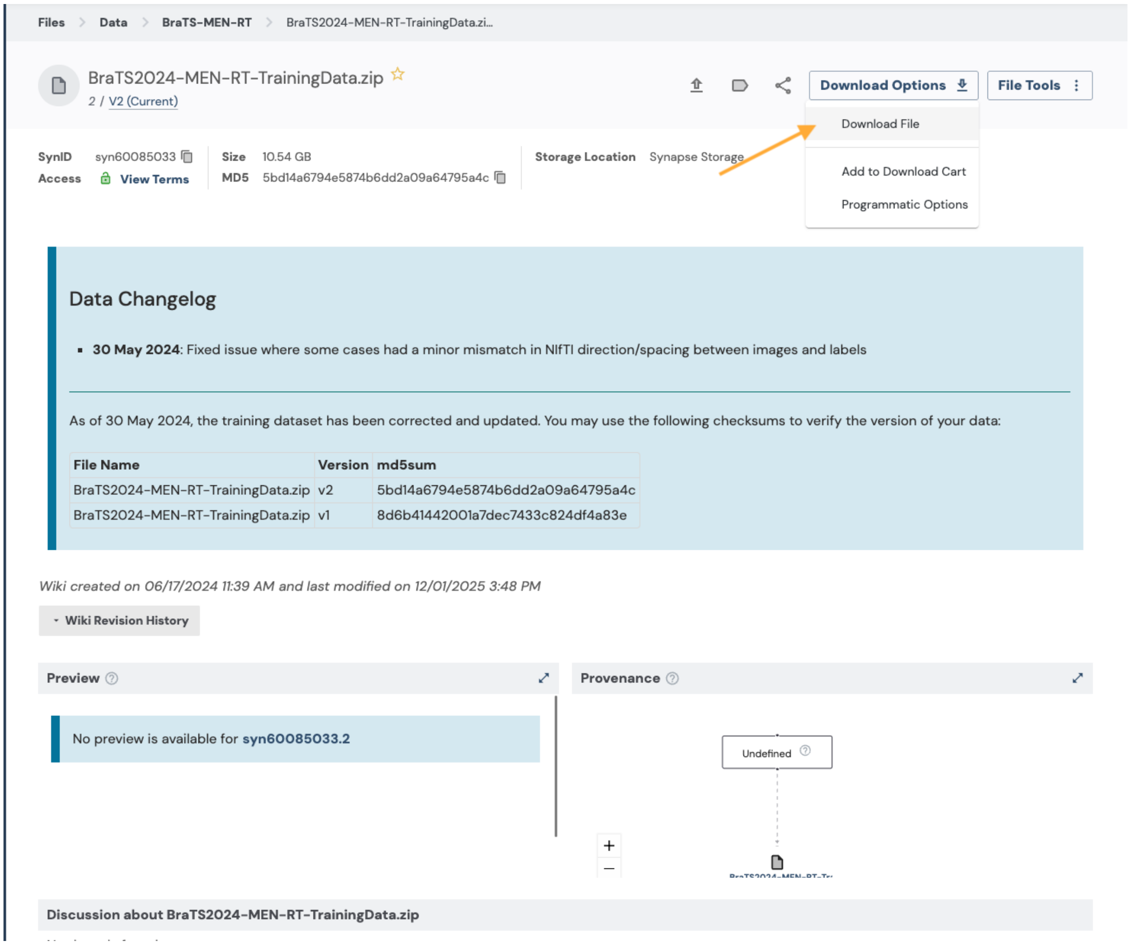

Option 3:

Users can also use the synapse CLI to programmatically download all the files in the folder, which could be more ideal as the CLI is much faster than the web UI counterpart, and takes advantage of multithreading to upload/download. To download:

1. Download the synapseclient (ideally into a python environment like conda or pyenv):

   ```
   pip install synapseclient
   ```

2. Authenticate yourself, using a Synapse Personal Access Token (PAT) that has "View", "Download" and "Modify" permissions:

   ```
   synapse config
   ```

   Follow the prompts to enter your username and authtoken.

3. Run the following:

   ```
   synapse get -r syn59059779
   ```
